# The Evolution of the Neural Basic Helix-Loop-Helix Proteins

**DOI:** 10.1100/tsw.2001.68

**Published:** 2002-04-23

**Authors:** Michel Vervoort, Valerie Ledent

**Affiliations:** Evolution et Développement des Protostomiens, Centre de Génétique Moléculaire-UPR 2067 CNRS, 1, av. de la terrasse, 91198 Gif-sur-Yvette Cedex, France; Belgian EMBnet Node - Bioinformatics Laboratory, Université Libre de Bruxelles, Department of Molecular Biology, Rue des Professeurs Jeener et Brachet 12, B-6041 Gosselies, Belgium

**Keywords:** basic Helix-loop-Helix (bHLH), nervous system, neurogenesis, evolution, neural specification, neural determination

## Abstract

Basic Helix-Loop-Helix (bHLH) transcription factors control various aspects of the formation of the nervous system in the metazoans. In Drosophila some bHLH (such as the achaete-scuteatonal, and amos genes) act as proneural genes, directing ectodermal cells toward a neural fate. Their vertebrate orthologs, however, probably do not assume such a neural determination function, but rather control the decision made by neural precursors to generate neurons and not glial cells, as well as the progression of neuronal precursors toward differentiation into mature neurons. The proneural function of Drosophila bHLH genes may be an innovation that occurs in the evolutive lineage that leads to arthropods. In addition, although neural bHLH appear to be involved in the specification of neuronal identities, they probably do not confer by themselves neuronal type-specific properties to the cells. Rather, neural bHLH allow neural cells to correctly interpret specification and positional cues provided by other factors. Although bHLH genes are often expressed in complementary subsets of neural cells and/or expressed sequentially in those cells, the coding regions of the various neural bHLH appear largely interchangeable. We propose that the specific expression patterns have been acquired, following gene duplications, by subfunctional-ization, i.e., the partitioning of ancestral expression patterns among the duplicates and, by extension, we propose that subfunctionalization is a key process to understand the evolution of neural bHLH genes.

## BHLH PROTEINS IN NERVOUS SYSTEM DEVELOPMENT

Proteins that contain a basic Helix-Loop-Helix (bHLH) domain are known in most eukaryotic clades. The bHLH domain is approximately 60 amino acids long and comprises a DNA-binding basic region followed by two a-helices separated by a variable loop region (HLH)[1]. The HLH domain promotes dimerization, allowing the formation of homo- or heterodimeric complexes between different family members[1]. The two basic domains brought together through dimerization bind specific DNA

hexanucleotides sequences and, accordingly, proteins containing this domain (referred as to bHLH proteins) have been shown to act as transcriptional regulators[1].

The bHLH proteins form a particularly large and complex family. We recently made an extensive search for bHLH proteins in sequence databases, including the completely sequenced genomes of Caernorhabditis elegans and of Drosophila[2,3] and found 35 different ones in C. elegans and 56 in Drosophila[4]. A phylogenetic analysis performed on these sequences, together with a large number of bHLH (>350 in total, including 90 characterized mouse bHLH) from several species, led us to define 44 orthologous families[4], among which 36 included bHLH from animals only, and 2 had representatives in both yeasts and animals. Fig. 1 shows a phylogenetic tree depicting these different families and their evolutionary relationships. In this review, we will focus on the bHLH which are involved in neural development and which belong to the families highlighted in Fig. 1. A nonexhaustive list of these genes can be found in Table 1 with some references.

Studies on neural bHLH have led to a general model about their function[5,6,7,8,9,10,11,12]. We first briefly summarize the principal assumptions of this model (Fig. 2).

1.As background to the model, it is believed that the generation of neural cells in both protostomes and deuterostomes involves a defined and evolutionary-conserved set of steps, each of which depends on the function of a small battery of evolutionary-conserved genes[5,6,12,13,14,15]. The earliest step consists in the commitment of more or less naive ectodermal cells to a neural precursor fate, the last step consists in the differentiation of the precursors or their progeny into functional nervous cells. Between these two steps, neural precursors may pass through intermediate stages and often divide and produce specific lineages. Commitment of ectodermal cells to a neural fate is first reversible (competence) and is given to a set of cells larger than the number of cells that eventually give rise to neural structures. Then, commitment becomes irreversible (determination) in a subset of the initially competence cells. The transition between the two states involves a cellular communication system (lateral inhibition)[16,17,18].

2.bHLH proteins act at the different steps mentioned above and are key elements for the realization of these steps.

3.Some of the bHLH genes are involved in early steps (determination events) but not in the late steps (differentiation) while others are involved in differentiation but not in determination. Early-acting genes have been named proneural (or neuronal determination) genes[7,19,20] to highlight their capability to direct the cells expressing them toward a neural fate. Late-acting genes have been named differentiation genes as they promote differentiation of already committed cells. In addition, still other bHLH are involved in less well-defined intermediate stages, between the initial determination and the terminal differentiation.

4.bHLH proteins do not only confer generic neuronal properties, but also neuronal type-specific properties, so that at the time they are born, neural precursors are not only committed to a neural fate but are, in addition, already committed to form (a) particular type(s) of neurons (a process often called neuronal specification). Both events (neural determination and neuronal specification) are tightly linked as both are controlled by the same bHLH factors.

5.bHLH proteins act in cascade, i.e., early-acting bHLH activate later-expressed ones, from the initial proneural genes to the late differentiation genes, through one or several intermediates. The organization in cascades is often presented as a kind of generic property of bHLH, mainly due to existence of similar cascade of bHLH proteins during myogenesis[21,22].

In this review, we discuss the above model in the light of recent data on the expression and function of neural bHLH in several developmental systems. We suggest that, contrary to their Drosophila

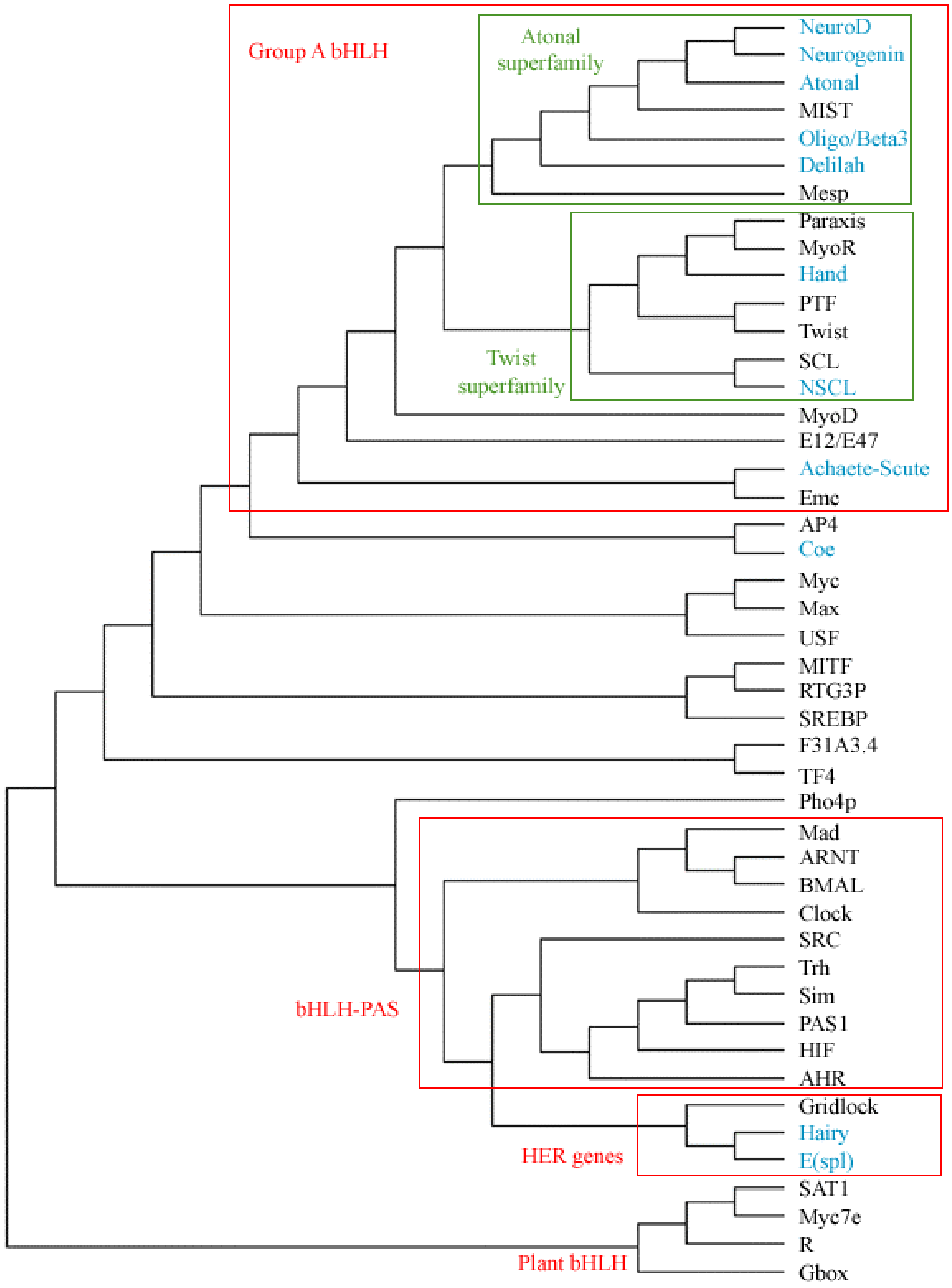

**FIGURE 1.** The 44 bHLH families and their evolutionary relationships**.** A NJ tree constructed using one gene (usually from mouse) per family, as well as the orphan D. melanogaster delilah gene and F31A3.4 (as a representative of a group of 3 C. elegans genes that cluster toghether with high bootstrap value). Families have been named according to their first discovered member, or in a few cases, to their best-characterized member[4]. To clearly represent the relationships at the tip of the branches, we display a rooted tree where the four plant bHLH families where taken as outgroup. This tree is just a representation of an unrooted tree with rooting that should be considered as arbitrary. For similar sake of simplicity, we show a tree in which branch lengths are not proportional to distances between sequences. Additional information can be found in Ledent and Vervoort[4] and on our web site *http://www.cnrs-gif.fr/cgm/evodevo/bhlh/index.html* . Families that contain proteins discussed in the review are coloured in blue. High-order grouping of the bHLH families is indicated. Group A includes related bHLH with similar biochemical properties, most of which being grouped into two superfamilies, Atonal and Twist. Other high-order groups are indicated such as bHLH-PAS, HER proteins and bHLH from plants**.** See Ledent and Vervoort[4] for details.

**TABLE 1**
**List of the bHLH Genes Described in the Review***

| GENES | SPECIES | FAMILY | REF. |
|---|---|---|---|
| *atonal* | DROME | ATONAL | [136] |
| *amos* | DROME | ATONAL | [139,140] |
| *cato* | DROME | ATONAL | [64] |
| *MATH1* | MOUSE | ATONAL | [132] |
| *XATH1* | XENLA | ATONAL | [59] |
| *MATH5* | MOUSE | ATONAL | [114] |
| *CATH5* | CHICK | ATONAL | [121] |
| *ZATH5* | BRARE | ATONAL | [122] |
| *XATH5* | XENLA | ATONAL | [58] |
| *lin-22* | CAEEL | ATONAL | [169] |
| *ASC* | DROME | ACHAETE-SCUTE | [16,36,37] |
| *JCASH1* | JUNCO | ACHAETE-SCUTE | [175] |
| *TcASH* | TRICA | ACHAETE-SCUTE | [175] |
| *MASH1* | MOUSE | ACHAETE-SCUTE | [68,69,75] |
| *CASH1* | CHICK | ACHAETE-SCUTE | [121] |
| *CASH4* | CHICK | ACHAETE-SCUTE | [46] |
| *XASH3* | XENLA | ACHAETE-SCUTE | [44,45,56] |
| *X-ngnr-1* | XENLA | NEUROGENIN | [51,53] |
| *tap* | DROME | NEUROGENIN | [161] |
| *M-ngn1* | MOUSE | NEUROGENIN | [51,77,78] |
| *M-ngn2* | MOUSE | NEUROGENIN | [51,77,79] |
| *C-ngn1* | CHICK | NEUROGENIN | [72] |
| *C-ngn2* | CHICK | NEUROGENIN | [72] |
| *Z-ngn* | BRARE | NEUROGENIN | [145] |
| *X-neuroD* | XENLA | NEUROD | [52] |
| *M-neuroD* | MOUSE | NEUROD | [52] |
| *C-neuroD* | CHICK | NEUROD | [121] |
| *NeuroM* | CHICK | NEUROD | [113] |
| *XATH3* | XENLA | NEUROD | [55,56] |
| *MATH3* | MOUSE | NEUROD | [70] |
| *XATH2* | XENLA | NEUROD | [57] |
| *cnd-1* | CAEEL | NEUROD | [168] |
| *CG11450* | DROME | NEUROD ? | [164] |
| *XCOE2* | XENLA | COE | [60] |
| *ZCOE2* | BRARE | COE | [156] |
| *M-oligo* | MOUSE | BETA3 | [102,103] |
| *M-HES1* | MOUSE | HER | [115] |
| *M-HES2* | MOUSE | HER | [119] |
| *M-HES3* | MOUSE | HER | [7,120] |
| *M-HES5* | MOUSE | HER | [118] |
| *M-HES6* | MOUSE | HER | [116,117] |
| *X-ESR1* | XENLA | HER | [7,120] |
| *X-ESR2* | XENLA | HER | [7,120] |
| *E(spl)-C* | DROME | HER | [120] |
| *deadpan* | DROME | HER | [159] |
| *dHand* | CHICK | HAND | [150] |
| *eHand* | CHICK | HAND | [150] |
| *NSCL-1* | MOUSE | NSCL | [157] |
| *NSCL-2* | MOUSE | NSCL | [158] |
| *C-NSCL-2* | CHICK | NSCL | [72] |

*Gene names are abbreviated as in the text. The Drosophila achaete-scute (ASC) and Enhancer of split (E(spl)-C) complexes include 4 and 7 related genes, respectively. M-oligo corresponds to three related genes (-1, -2 and -3). A more complete list of bHLH genes can be found in Ledent and Vervoort[4]. Species abbreviations are as follows: DROME = Drosophila melanogaster; CAEEL = Caenorhabditis elegans; MOUSE = Mus musculus; CHICK = Gallus gallus; XENLA = Xenopus laevis; BRARE = Brachydanio rerio; TRICA = Tribolium castaneum; JUNCO = Juonia coenia. Family assignment is based on phylogenetic reconstructions[4]. Additional information can be found on our web site http://www.cnrs-gif.fr/cgm/evodevo/bhlh/index.html.

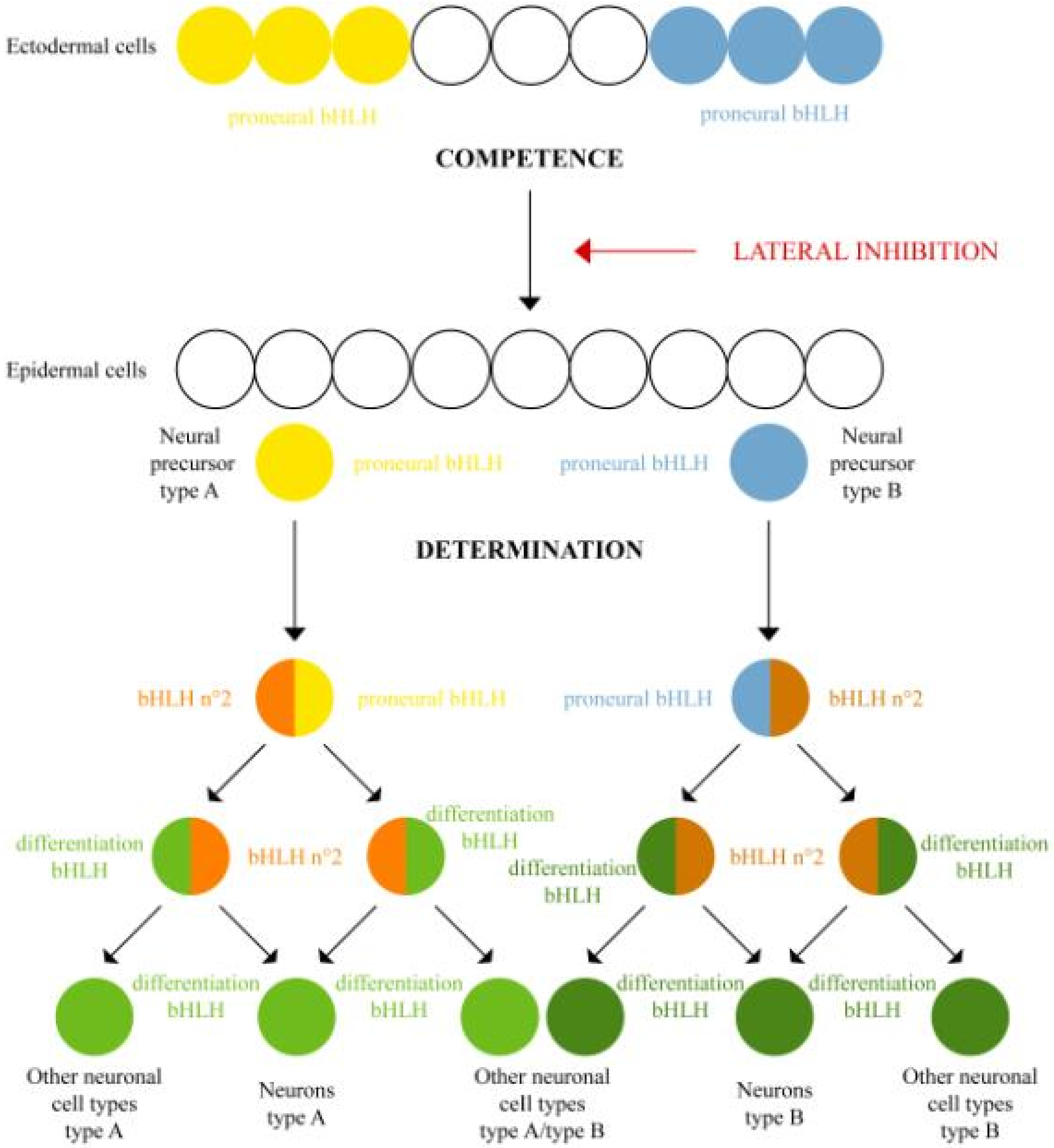

**FIGURE 2.** A general model for the function of bHLH during neural development. Some ectodermal cells express one specific bHLH (proneural bHLH) and acquire the competence to become neural precursors. Among those cells, a few isolated ones become irreversibly fated as neural precursors (determination) while most of the other revert to an epidermal fate by virtue of lateral inhibition. Neural precursors usually delaminate from the ectoderm and follow specific and reproducible lineages. Depending on the proneural bHLH expressed, neural precursors are committed to produce particular types of neurons (here mentioned as type A and B). Several bHLH are expressed during these lineages, in an overlapping way, from the proneural toward the differentiation bHLH. In the figure, only one intermediate (bHLH n°2) is shown but there may be more. The intermediate and differentiation bHLH found in the A and B lineages are shown as different genes but they may be identical.

counterparts that are involved in neural vs. epidermal fate decisions (proneural function), vertebrate early-acting bHLH control neuronal vs. glial cell fate choices in already committed neural precursors and/or the progression of these precursors toward differentiation. We further suggest that the differential expression of bHLH genes in specific neural precursors is not instrumental in defining the neuronal types generated by these precursors. Rather, we propose that the bHLH genes allow neural precursors and/or their progeny to correctly interpret specification and positional cues provided by others factors. Finally, we suggest that the fact that the different bHLH genes are often expressed in complementary subsets of neural cells and/or expressed sequentially in those cells can be best explained by subfunctionalization[23,24] and, by extension, we propose that subfunctionalization is a key process to understand the evolution of the neural bHLH proteins.

## PRONEURAL bHLH GENES

The concept of proneural gene was first defined in Drosophila and referred to the genes that are responsible for the decision made by naive ectodermal cells to adopt a neural fate[16,19,20]. These genes have the following properties. A complete loss-of-function (LOF) mutation of a proneural gene, i.e., a mutation which inactivates the gene, leads to a loss of neural structures, as a consequence of the conversion of neural precursors into epidermal cells. On the contrary, a gain-of-function mutation (GOF), i.e., a mutation which induces an over- or misexpression of the gene, leads to an excess of neural structures, as a consequence of the conversion of epidermal cells into neural precursors. The proneural genes are furthermore expressed at the time the neural precursors do form, their expression starting before any neural commitment has occurred. Neural determination, in Drosophila, is marked by the acquisition of specific cell properties, such as the delamination from the ectodermal epithelium and an altered cell cycle, as well as by the expression of a set of genes named neural precursor genes, such as scratch, prospero, and deadpan[25]. After delamination, the neural precursors follow a fixed lineage to produce various neurons, support cells, and glia[26,27,28,29,30,31,32,33,34,35].

A large set of experimental data indicates that five Drosophila genes display these properties: three of the four ASC genes (achaete, scute, and lethal of scute), atonal, and amos[6,8,12,36,37]. The question we ask here is whether or not there are proneural genes in vertebrates. If one reads the literature on the subject, one would surely answer yes to this question. In most recent reviews, and in a large number of research articles, three sets of vertebrate genes — ATH1 and ATH5 (atonal-related), neurogenin (ngn), and ASH (achaete-scute-related) genes — are presented as proneural genes[5,6,7,8,9,10,11,12]. Three main lines of argumentation have been used to define these vertebrate bHLH genes as proneural genes. First, they are expressed in early stages of nervous system development (and possibly at the time of or even prior to neural precursor determination). Second, knockouts of these genes in mouse lead to a more or less extensive loss of neural structures. Third, misexpression of these genes (mainly in frog) leads to the appearance of extra neural structures. Thus, these vertebrate bHLH seem to correspond perfectly well to the three criteria used in Drosophila to define a proneural gene, and one may conclude that the vertebrate genes cited above are bona fide proneural genes.

Our initial question may be clarified in the following way: do the available experimental data support a role for vertebrate bHLH genes qualitatively similar to that of Drosophila proneural genes, i.e., as factors controlling the commitment of ectodermal cells into neural precursors? To answer this question, we have to address more precisely the following points:

1. Are the vertebrate proneural genes expressed in naive ectodermal cells before their commitment as neural precursors?
2. What is the cellular basis of the loss of neural tissue observed in knockout mice?
3. What is the exact signification of the extra neurogenesis induced by misexpression experiments?

We will address these questions in four model systems which have been extensively characterized: 1) the formation of the neural plate and the primary neurogenesis in frog and zebrafish, 2) the formation of sensory and autonomic neurons from the neural crest, 3) the formation of the retina, and 4) the formation of the sensory elements of the inner ear from the otic placode.

## THE FORMATION OF THE NEURAL PLATE AND THE PRIMARY NEUROGENESIS IN FROG AND ZEBRAFISH

The embryonic nervous system of frog and zebrafish results from inductive interactions between the mesoderm and the ectoderm[38]. As a consequence of these interactions, a part of the ectoderm thickens and forms the neural plate. The neural plate eventually forms a cylindrical tube that subsequently differentiates into the central nervous system (CNS), through the fusion of its lateral margins in the frog or by cavitation in the zebrafish. Several successive rounds of neurogenesis do subsequently occur, generating a large diversity of neurons and glial cells. It is widely believed that all the cells of the early neural plate contribute to some extent to the neural tube and/or the neural crest[38]. In this view, neural determination would be concomitant with neural plate formation and all neural plate cells would be committed to a neural fate. There is no evidence that bHLH genes may be involved in neural plate formation; the best candidates so far described for this process are the HMG-box Sox genes and the odd-skipped-related zinc-finger Zic genes[39,40,41,42]. Nevertheless, a recent fate map of the chick caudal neural plate indicates that some neural plate cells may contribute to the epidermis, indicating that some neural plate cells may not be irreversibly committed to a neural fate and that neural vs. non-neural fate choice may occur inside the neural plate[43]. Interestingly, a bHLH gene is expressed in the most caudal part of the neural plate both in frog (XASH3)[44,45] and in chick (CASH4)[46] and may be involved in the determination of the neural identity in this part of the neural plate. Further analyses will be required to assess whether this can be extended to other vertebrates.

In frog and zebrafish, an early wave of neurogenesis occurs in the posterior neural plate, giving rise to the primary neurons that mediate an early escape reflex[47,48]. The primary neurons are formed by progenitors organized into three longitudinal stripes on each side of the dorsal midline (Fig. 3): a medial (future ventral) domain made of neural precursors that will generate motor neurons, an intermediate domain made of interneurons precursors, and a lateral (future dorsal) domain made of precursors of spinal sensory neurons (the Rohon-Beard neurons) and progenitors of the neural crest[47,48,49,50]. One bHLH gene, X-neurogenin-1 (X-ngr-1), is expressed at early stages of the frog neural plate in three bilateral stripes that correspond perfectly well to the domains of primary neurogenesis (Fig. 3)[51]. X-ngnr-1 is expressed before neural differentiation, as its expression precedes that of other genes such as NeuroD and N-tubulin that are expressed in postmitotic differentiating neurons (Fig. 3)[48,51,52]. The function of X-ngnr-1 during primary neurogenesis has been assessed by GOF experiments in Xenopus. Injection of X-ngnr-1 RNA induces ectopic neuronal differentiation when injected into Xenopus embryos at two-cell stage and is able to promote neuron formation in cultured animal caps[51,53]. This convincingly shows that X-ngnr-1 is able to convert ectodermal cells initially fated to form epidermis into neurons and suggests that during normal neural plate development, X-ngnr-1 may endow cells that express it with a neuronal fate.

However, similar ectopic neurogenesis can be induced by injection of various other bHLH RNA, such as XASH3[45,54] and its chick ortholog CASH4[46], NeuroD[52] and relatives such as XATH3[55,56] and XATH2[57], XATH5[58], XATH1[59], and the highly divergent bHLH XCOE2[60]. Most of the bHLH mentioned above are, however, not expressed at the time neural precursors do form but only later, even, in the case of NeuroD at the time the neurons differentiate[52] and cannot be involved in neural determination. Similarly, transient expression in mouse P19 embryonic carcinoma cells of early- and late-expressed bHLH genes such as MASH1, NeuroD, ngn1, and relatives is able to convert these cells into differentiated neurons[61]. A similar situation is also observed in Drosophila in which two

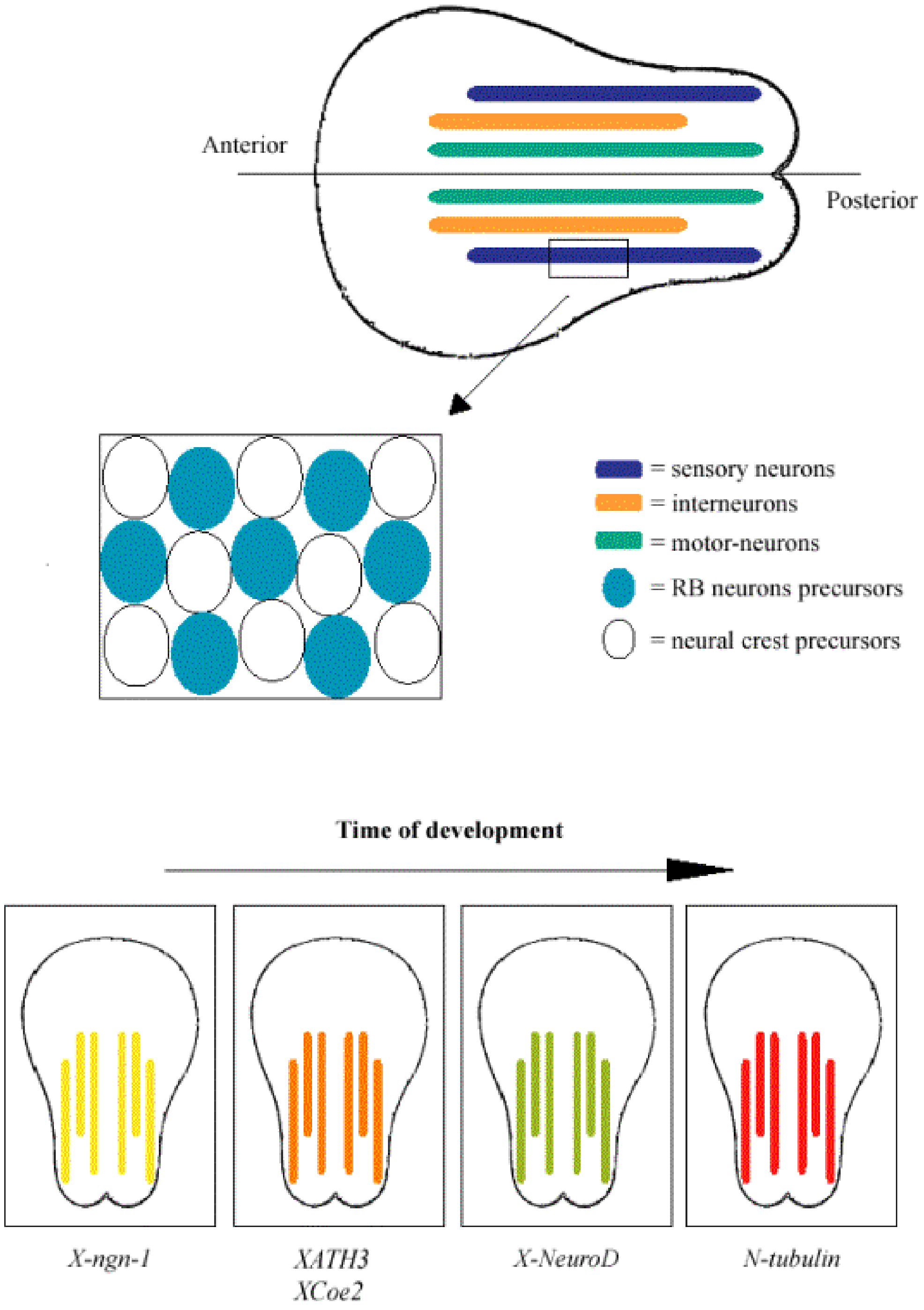

**FIGURE 3.** The primary neurogenesis in frog and zebrafish. (Upper part). Drawing of the neural plate depicting the three bilateral of primary neurogenesis. A portion of the lateral sensory stripe is highlighted in the insert. Two types of neural precursors are found, those of the Rohon-Beard (RB) neurons and premigratory neural crests precursors. (Lower part). Four successive stages (from left to right) of primary neurogenesis are depicted, showing some genes whose expression is initiated at that particular stage. For the sake of simplicity, only the initiation of the expression is shown. Nevertheless, when XATH3 and XCoe2 are first expressed, X-ngn-1 is still expressed. Similarly, XATH3 and XCoe2 expression overlaps that of X-NeuroD, which overlaps that of N-tubulin.

bHLH genes (asense and cousin of atonal [cato]), although expressed after neural precursors determination, are nevertheless capable to induce supernumerary neural structures[62,63,64].

This means that the capacity to elicit ectopic neurogenesis in GOF analyses is not per se indicative of the developmental role of a given bHLH. Furthermore, the induced ectopic neurogenesis may itself not be representative of the normal process: expression of bHLH genes such as MASH1 or ngn1 in cultured P19 cells induces their withdrawal from the cell cycle and their rapid differentiation into neurons[61] while in normal development these genes are expressed in cells that are still dividing and often produce complex lineages (see next section). Furthermore, overexpression (i.e., production of bHLH proteins at high nonphysiological levels) may overcome normal mechanisms (post-transcriptional and -translational regulations) that prevent or delay the function of these proteins. One such mechanism is known: the coexpression of the Id/Emc HLH proteins that lack a basic domain, prevents the function of various bHLH, through the formation of inactive heterodimers[1,16]. The formation of heterodimers is a stoichiometric process, thereby crucially relying on the relative quantity of the different proteins involved in this interaction. We therefore believe that the GOF results, altough proving the capacity of bHLH such as ASH and ngn genes to induce ectopic neuronal differentiation, do not provide strong evidence for a function in neural determination during normal development of the early neural plate.

## THE FORMATION OF SENSORY AND AUTONOMIC NEURONS FROM THE NEURAL CREST IN MOUSE AND CHICK

The neural crest gives rise to a large subset of the neurons and glia of the mouse and chick peripheral nervous system (PNS), including the trunk sensory neurons that innervate the skin and autonomic neurons that make part of the sympathetic, parasympathetic, and enteric nervous system (Fig. 4)[65,66]. In addition, numerous sensory neurons, such as those of the cranial ganglia, are produced by epidermal placodes that form in characteristic positions[67]. Several bHLH proteins are involved in the formation of sensory and autonomic neurons from the neural crest and the cephalic placodes (Fig. 4)[9], among which three have been proposed to be proneural genes, MASH1 and the two closely related neurogenin genes, ngn1 and ngn2[7,8,9,10,11].

MASH1 is expressed in the migrating precursors of the autonomic neurons while ngn1 and ngn2 are expressed in two complementary sets of precursors of both placode- and neural crest-derived sensory neurons in mouse and chick[51,68,69,70,71,72]. The exact timing of the expression of these bHLH genes is not completely clear with respect to the determination of the precursors. In the neural crest, the genes are not expressed in the premigratory precursors but are expressed in the early phases of the migration of these cells[70,71,72,73]. *In vitro* experiments provide indirect evidence that the earliest expression of ngn1 and ngn2 corresponds to the time where precursors become irreversibly fated as sensory neurons precursors[9,74]. Targeted mutagenesis experiments of ngn1, ngn2, and MASH1 have shown that these three genes are required for the formation of defined subsets of the PNS of the mouse[69,75,76,77,78,79].

In MASH1-null mice, most autonomic neurons do not form[69,75,76] or differentiate improperly[76,80]. Careful examination of the MASH1-null phenotype has shown that, in the absence of MASH1, multipotent neural crest stem cells are still committed to a neural fate but their further differentiation into mature neurons is blocked[75]. A similar phenotype is observed in the olfactory epithelium[69,81]. These observations strongly indicate that MASH1, at least during the formation of autonomic and olfactory neurons, is not involved in the commitment of ectodermal cells into neural cells but rather controls the correct differentiation of already determined neural precursors. Similarly, the analyses of the function of MASH1 in the retina and in the CNS (see below) argue against a role as proneural gene in the strict sense defined in Drosophila for its orthologs.

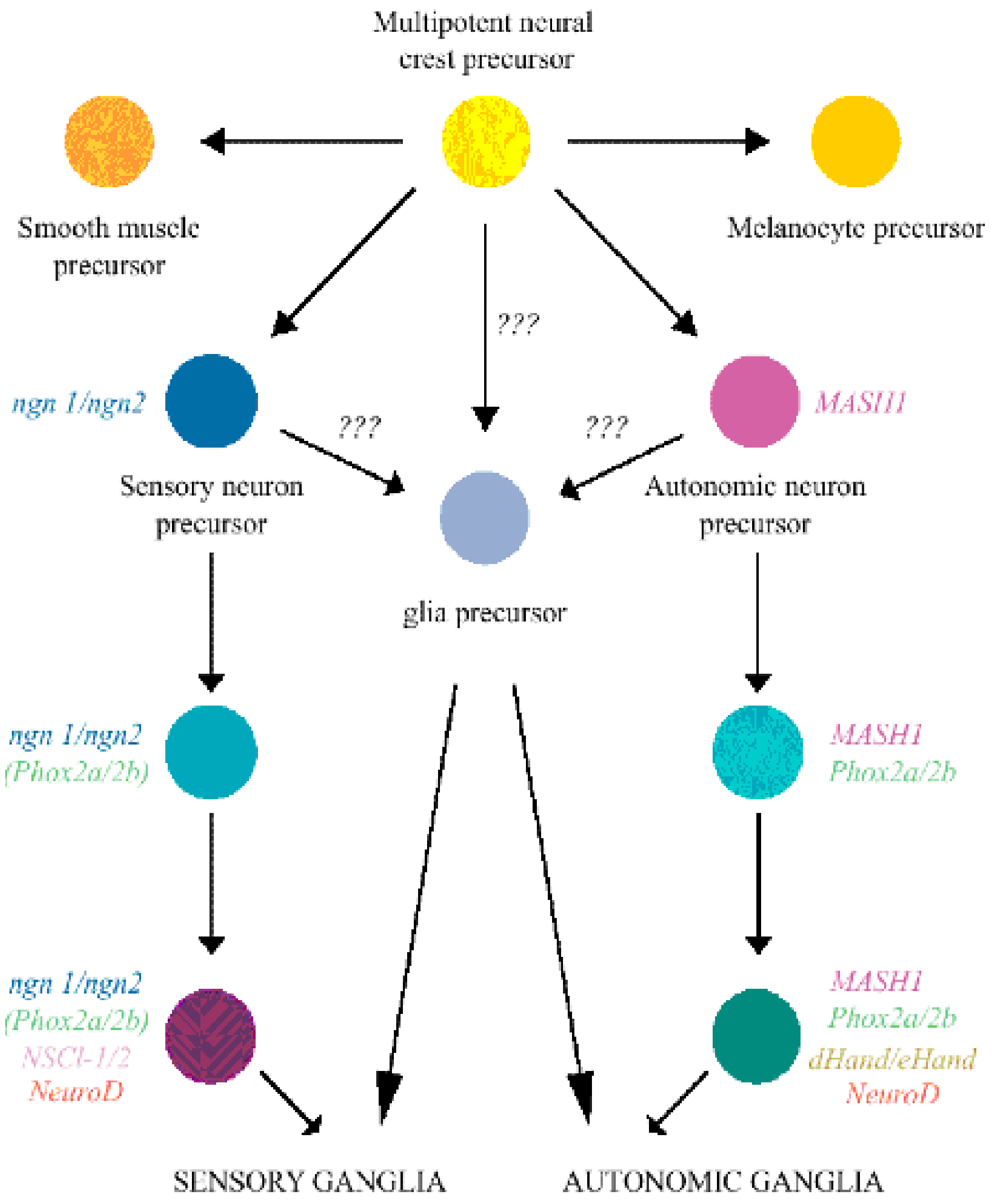

**FIGURE 4.** The formation of the autonomic and sensory ganglia from the neural crest. Multipotent neural crest precursors may adopt different fates. Precursor cells fated to generate sensory neurons express ngn genes; precursors fated to generate autonomic neurons express MASH1. They subsequently express other transcriptions factors such as Phox2a/2b (cells that will show a noradrenergic phenotype, i.e., all the MASH1- and a subset of the ngn-expressing precursors), NSCL1/2 (sensory neurons), dHand/eHand (autonomic neurons), and NeuroD (both types). These neurons contribute together with glial cells to the autonomic or sensory ganglia. Glial cells originate from glial precursors whose origin is uncertain; they may form a distinct subpopulation of precursors or may arise from sensory and autonomic neuron precursors (see text for details and references).

Knocking out in ngn1 or ngn2 genes prevents the formation of specific and complementary subsets of the sensory cranial ganglia that derived from the trigeminal and otic placodes[77,78,79]. To our knowledge, the developmental and cellular basis of the absence of sensory neurons in the mutant mice has not been directly assessed, i.e., it is not demonstrated whether the loss of neurons is due to the conversion of their precursors into epidermis (as shown for the proneural genes in Drosophila) or an impairment of their differentiation (as shown for MASH1 in the mouse PNS). We suggest that there exists a third possibility, for which there is compelling evidence, i.e., a conversion into glial cells. The sensory ganglia do not contain only neurons but also glial cells, both cell types being produced from multipotent neural crest precursors (Fig. 4). It has been shown that the differentiation of these two cell types is controlled by the Delta and Notch proteins[82,83] and may rely on an asymmetric cell division involving the Numb protein[83].

Notch is also involved in several other neural lineages where it controls glial vs. neuronal development[84,85,86,87,88,89]. A Notch/Delta/Numb system is similarly used in many asymmetric cell divisions that occur during neural development in Drosophila[90,91,92,93] and Notch has been shown to control neuronal vs. glial fate choices in some of these lineages[94,95]. These observations indicate that the absence of neurons observed in ngn-null mice may be, in principle, a consequence of their conversion into glial cells.

This suggestion deserves attention as it has been shown that ngn and other bHLH such as MASH1, MATH3, and NeuroD do actually control neuronal vs. glial cell fate determination in the retina (see next section) and in the CNS[96,97,98,99,100]. Knocking out in MASH1 or MATH3 induces rather moderate loss of neurons in the CNS[69,97,101]. In single MATH3-null mice, for example, the moderate loss of neurons that is observed is due to the cell death of committed neural precursors[97]. In double MASH1/MATH3 mutants, however, a severe loss of neurons is observed in the tectum, hindbrain, retina, and cells, which would otherwise differentiate into these neurons, then produce glia[97]. Similarly, ngn genes are able to suppress gliogenesis in the cerebral cortex by preventing the expression of glia-promoting factors such as the STAT transcription factor[99]. Furthermore, in mice mutant for both ngn2 and MASH1, an excess of cortical glia (astrocytic precursors) is observed at the expense of neuronal cells[100]. Taken together, these observations raise the possibility that MASH1 and ngn may be primarily involved in neural vs. glial cell fate choices. In single mutants, due to a partial redundancy with other bHLH such as MATH3 and NeuroD, the cells could be confronted with conflicting identities that may eventually lead to their incomplete differentiation and their death. In mice deficient for several bHLH, the full phenotype, i.e., conversion of neurons into glial cells, is observed.

Interestingly, a new family of bHLH (encoded by the oligo genes) controlling oligodendrocyte development has been recently characterized[102,103,104], suggesting that neuronal vs. glial development may be related to the expression of neuronal bHLH (such as MASH, MATH, and ngn genes) vs. the expression of glial bHLH (such as the oligo genes). However, the situation may be actually more complex: retrovirus-mediated misexpression of various bHLH such as MASH1, ngn, or MATH3 in mouse inhibits gliogenesis in the retina but not in the cerebral cortex where it induces neural degeneration[105]. Furthermore, oligodendrocytes that are found in the rat optic nerve express both MASH1 and oligo genes[102,103,106], indicating that a glial lineage may express both "neuronal" and "glial" bHLH.

Then, if MASH1, ngn1, and ngn2 are involved in neuronal vs. glial cell fate choice, are they proneural genes? We do not want to enter a semantic discussion about whether or not glial cells are neural or non-neural cells. What we want to stress is that in Drosophila, the proneural genes promote the formation of precursors that generate both neurons and glial cells[26,27,28,29,30,31,32,33,34,35]. In the absence of Drosophila proneural genes, both glial and neuronal cells are absent due to their conversion of their common precursor into an epidermoblast that will eventually differentiate into an epidermal cell. If MASH1, ngn1, and ngn2 do actually control neuronal vs. glial cell fate choices (both of which making part of the nervous system), then their developmental function differs from that of the Drosophila proneural genes (neural vs. epidermal fate).

## THE FORMATION OF THE VERTEBRATE RETINA

During vertebrate development, the retina evaginates from the neural tube and forms a relatively simple laminated tissue. Seven distinct neural types originate from this simple neuroepithelial sheet of cells: six types of neurons (retinal ganglion cells, amacrine cells, bipolar cells, horizontal cells, rod and cone photoreceptor cells) and one type of glial cells (Müller cells)[107]. These cell types are formed in an orderly manner from multipotent progenitors[107]. Ganglion, cone photoreceptor, and horizontal cells are born first, followed by amacrine and rod photoreceptor cells, while bipolar and Müller cells are born last[107]. Cell fate allocations use both extrinsic and intrinsic cues[108], among which is the expression of several bHLH genes, ASH genes[96,109,110,111], ngn genes[70,112], NeuroD and relatives[98,113], and ATH5[58,111,114]. Several HES bHLH genes are also expressed in the retina[115,116,117,118,119]. HES proteins (also sometimes called ESR or HER proteins) and their orthologs in Drosophila (e.g., Hairy, deadpan, Hey, and the Enhancer of split genes) form a monophyletic group of bHLH (Fig. 1)[4] — the HER family — with specific biochemical features[120]. The HER proteins have been shown, in many instances, both in Drosophila and vertebrates, to suppress neural development by counteracting other bHLH proteins[7,120], although one of them, at least, HES6, promotes neuronal development in the retina[116,117]. Several HER proteins act as effectors of Notch/Delta signal and are involved in the same processes[7,120].

A large body of work has been done on the ATH5 genes (atonal orthologs, see Table 1), in Xenopus[58,111], mouse[114], chick[121], and zebrafish[122]. In these four organisms, ATH5 is expressed early in retinal neurogenesis, probably by retinal cells progenitors before these cells differentiate[58,111,114,122]. In mouse, the expression of MATH5 precedes that of all other bHLH expressed in the retina[114]. In chick, however, CATH5, C-ngn-2, and CASH1 are expressed at about the same time - CATH5 and C-ngn-2 in the same subset of retinal cells, and CASH1 in a complementary subset of cells (Fig. 5)[121]. In this case, it has been suggested that ATH5 and ngn are expressed in precursors that will generate early-born retinal cells (e.g., retinal ganglion cells) while CASH1 is expressed in precursors that will generate later-born retinal cells (such as amacrine and bipolar cells) (Fig. 5)[114,121]. Targeted deletion of MATH5 blocks the differentiation of most retinal ganglion cells and results in an increase of differentiated amacrine cells and Müller glia[123]. Overexpression of XATH5 in frog leads to the opposite defects (an excess of ganglion cells and a lack of amacrine cells and Müller glia)[58]. Together, these observations indicate a key function for ATH5 in the determination of retinal ganglion cells (early-born neurons) vs. other later-born retinal cell types (including Müller glia) but not in the determination of a neural fate vs. non-neural fate. Similarly, overexpression of X-ngnr-1 or XATH3 in frog retina induces a loss of late-born retinal cells and an excess of early-born cells, photoreceptor cells with X-ngnr-1 and both photoreceptor and ganglion cells with XATH3[56].

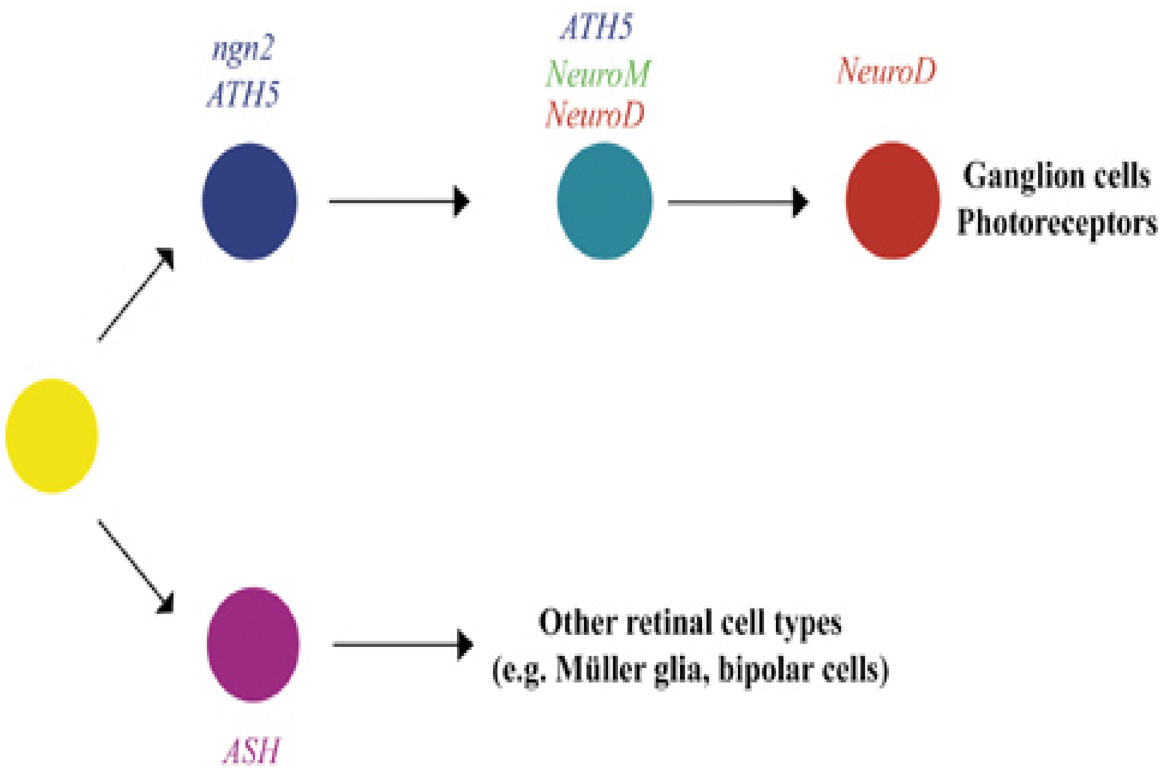

**FIGURE 5.** The determination of neural cell types in the retina. Multipotent precursor cells may express either ngn2 and ATH5 or ASH1. ngn2- and ATH5-expressing precursors subsequently express NeuroM/ATH3 and NeuroD and eventually differentiate into ganglion cells and photoreceptors. ASH1-expressing precursors differentiate later in development other retinal cell types such as Müller glia and bipolar cells.

Similar observations have been made for NeuroD and ASH genes and point out a function for these genes in the neuron vs. glial cell fate decision and in neural differentiation and/or survival[108]. Indeed, LOF of these genes leads to an excess of Müller glia while their overexpression induces the almost complete loss of this cell type[58,96,97,98,124]. A fate transformation of Müller glia into bipolar cells was clearly observed in mice mutant for both MASH1 and MATH3, indicating that, as in the CNS, the absence of both genes leads to the conversion of neuronal into glial cells[97]. Overexpression of either of these genes in mouse retina does not, however, promote bipolar cell genesis, but does inhibit Müller gliogenesis[124]. In addition to the expression of the bHLH genes, the formation of extra bipolar cells requires the expression of the homeobox gene chx10[124].

Taken together, all these data indicate that, in the retina, neural bHLH may be involved in the choice between alternative neural cell fates (particularly between glia and neurons) and/or differentiation of these cells rather than in neural/non-neural cell fate choices, as their Drosophila counterparts.

## THE FORMATION OF THE SENSORY ELEMENTS OF THE VERTEBRATE INNER EAR FROM THE OTIC PLACODE

The inner ear in vertebrates is made of several distinct mechanosensory structures that allow the sense of sound vibrations, linear acceleration, gravity, and rotation. These structures contain a sensory epithelium made of mechanoreceptors (the hair cells) and their associated support cells (Fig. 6). In addition, sensory neurons (in particular cephalic ganglia) innervate the sensory epithelium and convey the information from the hair cells toward the CNS. All these cells originate from the same ectodermal placode, the otic placode[125]. During early otic placode development, neuroblasts delaminate and form the sensory neurons[126]. The otic placode then invaginates and becomes the otocyst from which the inner ear will develop. In the sensory epithelium of the inner ear, hair cells and support cells are usually more or less regularly interspersed, in such a way that individual hair cells are surrounded by supporting cells (Fig. 6)[126,127]. It is widely thought that hair cells and support cells are siblings, arising from common precursors[128] and that the determination of the two cell fates relies on lateral inhibition[126,129,130,131], in a way very reminiscent to that which happens during Drosophila sense organ lineages[90,91,92].

Three types of bHLH have been shown to have important functions during inner ear formation: ngn[77], MATH1[132,133,134], and NeuroD genes[135]. As already mentioned, knocking out in ngn1 or ngn2 genes prevents the formation of the sensory cranial ganglia that derive from the trigeminal and otic placodes[77,78,79]. As stated in the previous section, the developmental and cellular basis of the absence of these neurons in the mutant mice has not been described. Hence, a neuronal to glial cell fate conversion cannot be ruled out. MATH1 is expressed throughout the inner ear sensory epithelium and its expression becomes secondarily restricted to the future hair cells (Fig. 6)[132,133]. Embryonic MATH1-null mice fail to generate hair cells while support cells and sensory neurons do normally form[132]. Overexpression of MATH1 in mice induces the appearance of extra hair cells in the inner ear[134].

Taken together, these observations indicate that MATH1 is required for the formation of hair cells and may control the choice between hair cell and support cell fates. Finally, it has been recently shown that NeuroD is required for the differentiation and/or survival of the sensory neurons that innervate the inner ear but not for their generation, nor for the formation of the inner ear epithelium[135].

In conclusion, analysis of bHLH function performed on the inner ear of vertebrates does not provide reliable arguments in favour of a role of bHLH in neural/non-neural cell fate decision during otic placode development. Rather, bHLH genes may be involved in choices between alternative neural identities and for the survival of differentiated neural cells.

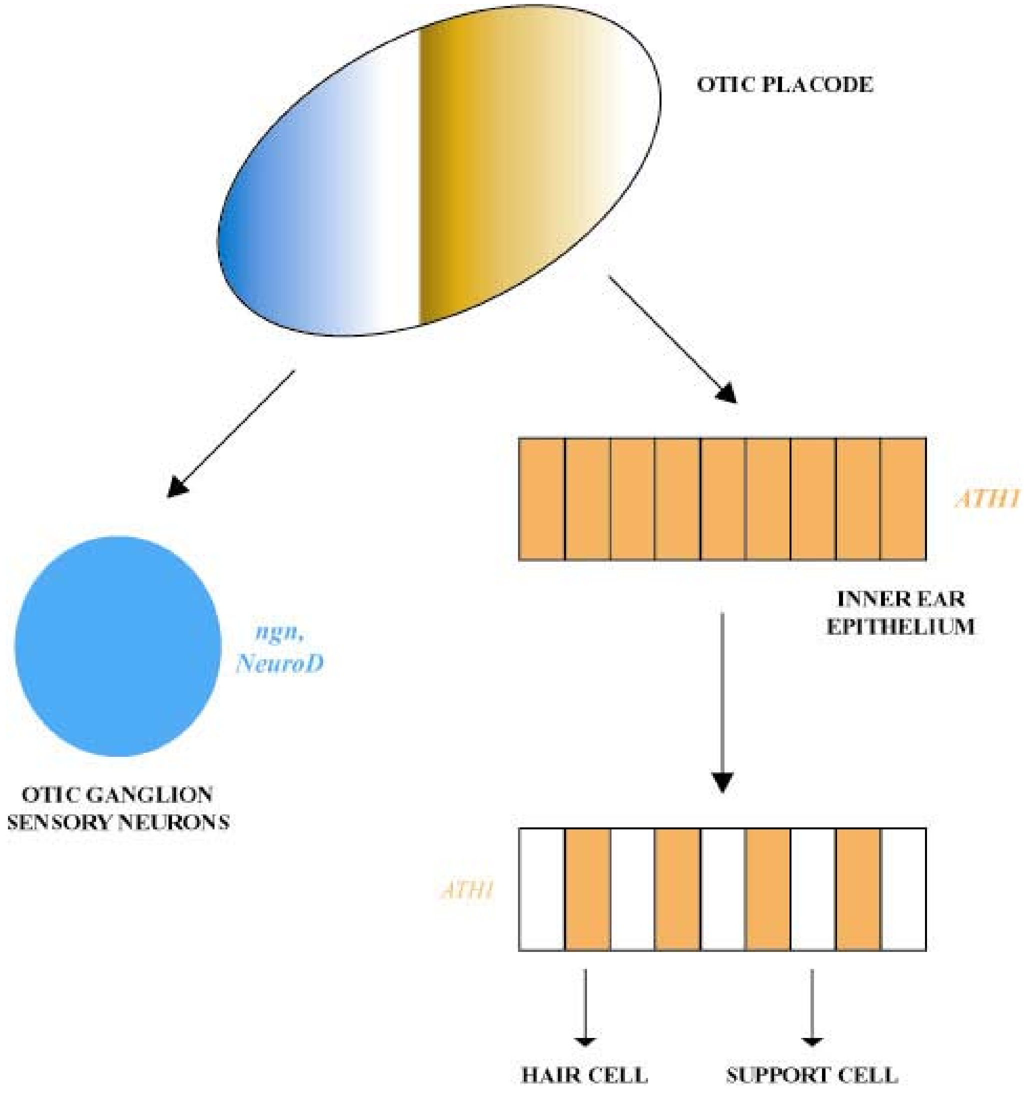

**FIGURE 6.** The formation of the inner ear sensory elements from the otic placode. The otic placode gives rise to both the otic ganglia (left) and to the inner ear sensory epithelia (right). The formation of the otic ganglia involves both ngn and NeuroD genes. The whole inner ear epithelium expresses first ATH1, then ATH1 expression becomes restricted to isolated cells that differentiate into hair cells. The interspersed cells that do not express ATH1 differentiate into support cells.

As a general conclusion of this section, we believe there is no clear evidence for a role for vertebrate bHLH genes comparable to that found in Drosophila, i.e., in the choices between neural and non-neural fates. Rather, vertebrate bHLH are involved in neuronal vs. glial choices, in the progression of committed neural precursors toward differentiation, and in the survival of the neural cells. The proneural function, as defined in Drosophila, may be a specific feature of Drosophila (and maybe C. elegans, see below).

## bHLH AS SPECIFICATION GENES

In the past few years, it has become increasingly clear that at the time a neural precursor is born, it is already fated to give rise to specific neuronal subtypes (by a process generally called neural specification). It also has been proposed that the neural bHLH confer not only generic neural properties to the cells but also subtype-specific properties, linking by this way neural determination and neuronal specification[6,9,11]. In other words, a same set of genes would control the determination of cells as neural precursors but also provide these cells with information about the types of neurons to produce. In the previous section, we summarized recent data that indicate that vertebrate bHLH genes control the formation of neurons vs. glial cells. In this section, we will discuss the (lack of) evidence that bHLH genes control the type of neurons that are produced by neuronal precursors.

The first apparently compelling evidence comes from the expression patterns of the neural bHLH genes. These genes are often expressed in complementary sets of cells that correspond to different types of neurons and/or neural structures. This is, for example, the case in the Drosophila PNS (Fig. 7). Three types of proneural bHLH are known to be required for complementary sets of sense organs[6,12,19,36,37]: ASC genes are required for sense organs that display external structures visible on the cuticle (often a bristle) and therefore called external sense organs (es organs); atonal for the completely internal mechanosensory organs, called chordotonal organs (ch organs), the photoreceptors of the eye and a subset of the olfactive sense organs of the antennae; and amos for the atonal-independent olfactive sense organs of the antennae. Similarly, in vertebrates, the three types of bHLH involved in early steps of neural development — ngn, ATH1/5, and ASH genes — are expressed in largely complementary subsets of the nervous system and, therefore, are required for different types of neural cells[9,10,11,12]. Given that these different bHLH are expressed in largely nonoverlapping sets of neural precursors that will give rise to different types of neural derivatives, it is tempting to think that these genes will not solely give generic neural properties to the precursors (why, in this case, have different genes that would have the same function?) but also confer upon them properties specific of the type of sense organs they will generate. We will, however, argue later in this review, following the model of subfunctionalization proposed by Force et al.[23], that to have several structurally related bHLH (i.e., that have been generated at some time in the past by duplications from a single ancestral genes) implies necessarily that they have different expression patterns but does not necessarily imply, however, that they have different functions.

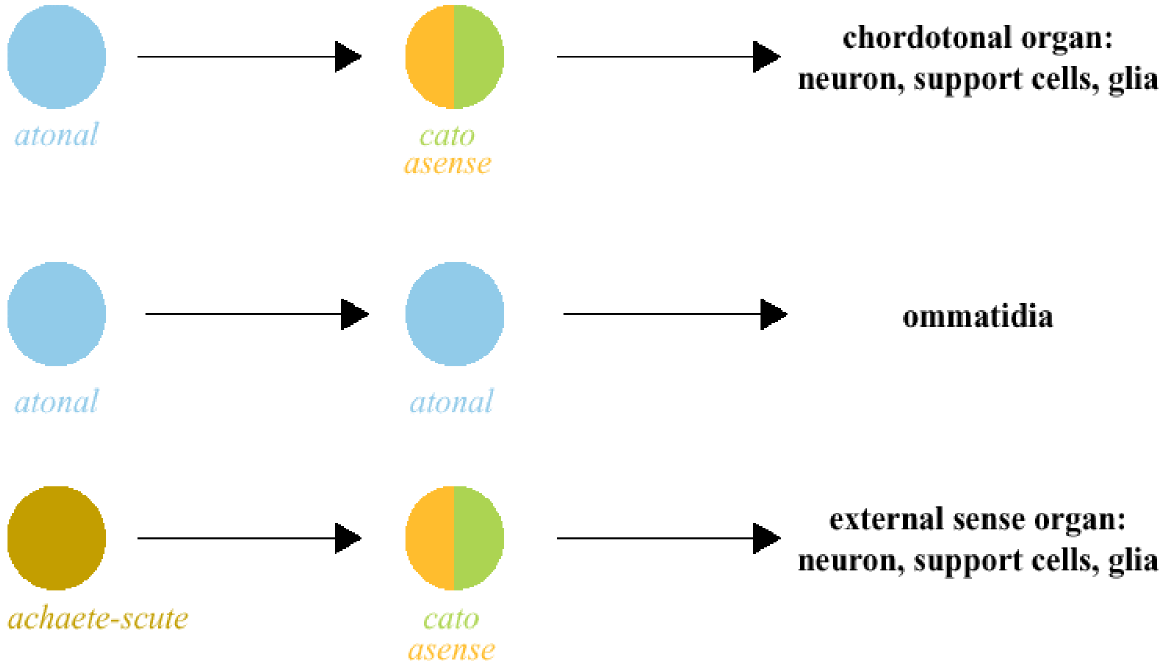

**FIGURE 7.** Proneural genes and sense organ specification in Drosophila. Sense organ precursors expressing atonal or achaete-scute genes are shown. Depending on where they do form, atonal-expressing precursors generate chordotonal organs (ch organs) or ommatidia. achaete-scute-expressing precursors generate external sense organs (es organs). Both es and ch organ precursors express neural precursors genes such as the bHLH genes cato and asense and generate the full set of cells constituting the sense organs, i.e., accessory cells, neurons and glia. The atonal-expressing cells in the eye become R8 photoreceptors that induced neighbouring cells to form the remaining 7 photoreceptors and several accessory cells constituting the ommatidia.

The second line of evidence for a role of neural bHLH in neural specification comes from GOF experiments conducted in Drosophila[8,11]. The initial observation is quite simple: if one expresses ubiquitously any one of the genes of the ASC at the time sense organ precursors are born, one will observe extra sense organs of the es type (the type normally promoted by the ASC genes) but never of the ch type (normally promoted by atonal). In contrast, ubiquitous expression of atonal induces both es and ch extra sense organs. The induction of es organs by atonal has been proposed to arise from artefactual activation of endogenous ASC as a result of the overexpression of atonal[136,137]. Accordingly, using specific conditions of misexpression of atonal, Jarman and Ahmed[138] obtained subtype specificity, i.e., induction of only extra ch organs. Furthermore, in these specific conditions, conversion of es organs into ch ones was sometimes observed[138]. The functional differences between atonal and ASC proteins were assessed by domain swapping exper-iments[137]. These experiments showed that the capability of atonal to induce extra ch organs in GOF experiments resides in the basic (b) region of its bHLH motif. The b regions of ato and of the ASC proteins differ by only seven residues that seem to project away from DNA in the ato protein, an observation that has lead to the idea that the ch vs. es specificity of the bHLH factor depends on an interaction of the b domain with cofactor(s)[137]. Taken together, these observations have been reported since then in many articles as argument to demonstrate that proneural proteins confer specificity to the precursors that express them[8,9,10,11,12].

However, we do not find this evidence so compelling. The ubiquitous expression of atonal induces supernumerary sense organs, 50% of them being of the es type[136,137], indicating that the induction of es organs is not a rare or marginal phenotype. Similarly, the third type of proneural proteins, amos, when ectopically expressed, is able to induce both es and ch ectopic organs, although this gene is normally not required for the formation of these organs[139,140]. Furthermore, the forced expression of one of the ASC genes, scute, in the eye (where it is normally not expressed) in an ato- background (where normally all photoreceptors are missing)[141] induces the formation of photoreceptors[142]. These observations do not particularly argue for a specificity of the proneural proteins. Nevertheless, ectopic expression of ASC genes does produce only es organs and, in some conditions, ectopic atonal expression induces only ch organs and even converts es into ch organs[138]. We think that these observations may be linked to the function of the homeobox gene cut. Cut is expressed in the ASC-dependent precursors and their progeny and is necessary and sufficient to confer es-specific properties to these cells, e.g., the capacity to produce external cuticular derivatives[25]. The expression of cut is dependent on the ASC genes, possibly in a direct way[25]. It is not surprising that ectopic expression of ASC genes elicits cut expression, thereby always generating sense organs with es properties. We suggest that atonal is also able to activate cut but less efficiently than ASC proteins, maybe due to minor evolutionary alterations that may have occurred in the cut regulatory regions. (It has become increasingly clear that there is a coevolution between transcription factors and regulatory regions[143].) When atonal is ectopically expressed at high level[136,137], it is conceivable that, depending on the exact quantity of atonal and of the cellular environment, atonal does or does not activate cut, in a more or less random manner, leading to formation of es or ch organs in a more or less random way. In experiments in which atonal is expressed at lower levels[138], the quantity of atonal may be always insufficient to activate cut, hence producing only ch organs. The presence of low quantity of atonal in es precursors may even, through binding to ASC proteins or to their indispensable cofactor, Daughterless, have dominant negative effects, thereby preventing the expression of cut in those es precursors, hence converting them into ch precursors. The implication of a gene in neuronal specification may, in theory, be highlighted by its LOF phenotype, as it may show a conversion of one neuronal subtype into another. This is for example, the case for cut: in cut-null mutants, es organs are converted into ch organs, i.e., cells that would have normally show es-specific properties display ch-like properties[25]. LOF analyses of Drosophila proneural genes, however, do not allow assessing their potential function in neural specification, as neural precursors are absent and therefore no neural structure is produced. Similarly, in vertebrates, in bHLH-knockout mice, subsets of neurons are often absent and potential neural identity conversion can not be assessed. For example, there is a good correlation between MASH1 expression and noradrenergic differentiation in mouse, suggesting that MASH1 may specify this neuronal type[11,144]. In MASH1-null mice, a large part of the noradrenergic neurons fail to form[11], indicating that MASH1 is required for the differentiation of neurons that will show a noradrenergic phenotype. This does not demonstrate, however, that MASH1 is responsible for the acquisition of the noradrenergic phenotype by these cells. Along that line, it is worth mentioning, as did Brunet and Ghysen[11], that some of the cells that express MASH1 do not differentiate noradrenergic neurons and that numerous cells that do not express MASH1 nevertheless display a noradrenergic phenotype (as for example, the ngn-dependent cranial sensory neurons). This example shows that it is not so obvious to correlate a defined bHLH with a particular precise neural fate.

One such correlation often mentioned is made between ASH genes with autonomic neurons and ngn genes with sensory neurons, in the neural crest[9]. In knockout mice, however, no conversion of one neuronal fate into the other one can be observed, the observed phenotype being the absence of neurons, maybe due to their conversion into glial cells (see previous section). Specificity has been assessed using GOF experiments. The most compelling evidence comes from experiments made on the chick neural crest. Chick migrating crest cells infected with a retrovirus that confers a constitutive expression of mouse ngn-1, are often found later in the sensory ganglia and only rarely in sympathetic ganglia, suggesting that expression of ngn biases migrating crest cells to adopt a sensory neuronal fate[72]. Furthermore, in the same assay, ngn-1 was able to induce sensory neurons markers in autonomic neurons and even in the dermomyotome, a mesodermal derivative[72]. Following these results, ngn could be involved in the specification of sensory neural properties and even specifies this particular cell fate. Alternatively, ngn may simply render neuronal precursors competent to respond to other factors that specify neuronal identity. In agreement, the injection of ngn RNA in early zebrafish embryos induces ectopic primary neurons whose identity depends on their dorso-ventral position and is influenced by Sonic hedgehog[145]. Similarly, in the zebrafish neural plate, the identity of the primary neurons as motor-, inter-, or sensory neurons depends on antagonistic Sonic hedgehog and BMP (bone morphogenetic proteins) signaling that occurred at the time of the formation of the neural plate[146].

In the neural crest, it is, furthermore, well known that neuronal identities are controlled by growth factors such as BMPs[66,73,147,148,149]. BMP2/4 have been shown to promote the formation of autonomic neurons[66,73,147,148], inducing the expression of transcription factors such as the paired homeodomain Phox2a and Phox2b and the bHLH dHand and eHand (Fig. 4)[147,148,150]. These proteins are expressed after ASH1 in the autonomic lineage in mouse and chick[150,151,152] and are able to elicit the formation of ectopic sympathetic neurons[150,153,154]. Phox2b has been further shown to be required for sympathetic ganglia formation and to control the noradrenergic phenotype of these neurons[144,151,155]. In this case, ASH1 may be mainly or even solely required to allow BMPs to activate other transcription factors that impose specific neural properties to the cells. It is worth mentioning that expression of different neural bHLH in mouse P19 embryonic cells converts them into neurons that express similar or identical patterns of neurotransmitters and other neuronal proteins[61].

Taken together, these observations suggest that neural bHLH do not control directly the specification of particular neuronal subtypes, but rather, that they may be required to allow the correct interpretation by the cells, of subtype information given by other factors[66,149].

## CASCADES OF SPECIALIZED BHLH PROTEINS

In vertebrates, several different bHLH are often expressed in a given lineage in successive stages of this lineage[9]. From this type of observation, it has been proposed that cascades[77,81] or networks[58] of bHLH may function during all stages of vertebrate neural development from commitment of precursors, through proliferation and migration[51,113], and finally to postmitotic terminal differentiation[52]. In such genetic cascades, bHLH proteins would control appropriate target genes of each of these stages, including the activation of the next bHLH protein in the cascade. The picture is similar to the cascade of bHLH involved in myogenesis and it has been often quoted that the existence of cascades may be a general feature of bHLH proteins[9,22]. There is no doubt that cascades of bHLH during neural development in vertebrates do exist. In this section, we will discuss to which extent this represents a general rule. Furthermore, we will also discuss the functional signification of these cascades, i.e., the existence of functional specializations of the bHLH proteins with respect to the stages they are acting.

In vertebrates, ngn and ASH genes are usually the first bHLH to be expressed in neural lineages and their expression is often found in complementary subsets of nervous cells (see previous sections; an exception is the lineage that leads to the olfactive neurons[81]). In most cases, the expression of ngn and ASH is followed by that of NeuroD-related genes, first the genes of the ATH3/NeuroM subfamily and then the NeuroD genes (Figs. 3, 4, and 5)[9]. ATH3/NeuroM expression overlaps those of ngn and ASH, and is found exclusively in undifferentiated (but already determined) cells. The expression of NeuroD genes overlaps that of ATH3/NeuroM and coincides with neural differentiation. Other bHLH are also expressed in some of these lineages, after ngn and/or ASH expression, such as Coe genes during primary neurogenesis in frog and zebrafish[60,156] (Fig. 3) and NSCL and Hand genes in neural crest derivatives[150,157,158] (Fig. 4). In many cases, the expression of early bHLH has shown to be both necessary and sufficient to activate late bHLH, highlighting the existence of genetic cascades[9].

It has also been suggested that bHLH cascades act during Drosophila sense organs formation[64]. The expression of the proneural genes — ASC genes (scute, achaete), atonal and amos — in ectodermal patches of cells allow the formation of neural precursors that express several "neural precursor genes"[25] among which are bHLH genes such as the ASC gene asense, the atonal-related gene cato, and the HER gene deadpan (Fig. 7)[62,63,64,159]. The expression of asense and cato is dependent initially on the proneural genes and is maintained after the proneural genes expression has ceased, until shortly before neuronal differentiation[62,63,64]. These observations have led to the idea that, as in vertebrates, there are cascades of bHLH proteins in Drosophila[64]. However, in at least one case, this does not hold true: photoreceptor development appears to only involve atonal and no other bHLH[64,136]. In this case, atonal appears to be involved both in the determination and the differentiation of the photoreceptors[160]. In vertebrates, the cascades of bHLH involve ngn and NeuroD genes. The Drosophila ortholog of the ngn genes, tap, is expressed in one particular lineage that will give rise to gustative sense organs, shortly before neural differentiation and its expression seems not to be specially related to that of other bHLH[161,162]. A putative Drosophila ortholog of the NeuroD genes (named CG11450) has been recently described, based on overall similarity[12]. The orthology of this gene to vertebrate NeuroD genes is, however, not supported by phylogenetic analyses[4]. Moreover, preliminary analyses of the expression of this gene suggest it is not involved in nervous system formation[163,164]. Taken together, these observations indicate that the existence of bHLH cascades similar to those of vertebrates, in Drosophila, is not particularly obvious.

Another failure to the idea that cascades are a general feature of bHLH comes from the nematode, C. elegans. The C. elegans nervous system contains 302 neurons representing 118 classes based on morphology, connectivity, and position[165]. The neurons are generated by invariant patterns of cell divisions and migrations[166]. Most (but not all) embryonic neuroblasts and their progeny express the *C. elegans* ortholog of Daughterless/E12, some of which also expressing an ASH-related bHLH[167]. No functional analysis of this ASH gene has been, so far, published and four additional uncharacterised ASH genes are found in the genome[4]. Nevertheless, its expression appears to cover the whole lineage of the neuroblasts that express it. A large set of the neuroblasts and their progeny also express the single *C. elegans* NeuroD gene, cnd-1[168]. A single atonal ortholog, lin-32, has also been cloned[169]. LOF mutants lack several neurons and sense organs formed during postembryonic development[169]. This includes the rays found in the male tail, i.e., small sensilla made of two neurons and a structural cell, which are used during mating[170]. The ray is produced by a sublineage of the posterior V seam cells that also produce specialized epidermal cells and other neuroblasts (Fig. 8)[171]. In brief, a ray precursor cell (Rn) gives rise to an anterior daughter (Rn.a, the ray neuroblast) that produce the ray and a posterior daughter (Rn.p) that differentiate into an epidermal cell. In lin-32 LOF mutants, the two daughters of Rn generate epidermal cells[169]. The expression of lin-32 during postembryonic development has been assessed using reporter fusions: lin-32 is expressed throughout the sublineage, from the Rn cells until the terminal division[172]. Genetic analyses involving hypomorphic mutations (where the function is not completely abolished) showed that lin-32 may be functionally important in all steps of the lineage: rays made only of neurons or only of structural cells are often observed[172]. These observations indicate that lin-32 is involved from early steps of the lineages, in neural determination (in a way that may be akin to that of Drosophila proneural genes) until late differentiation steps. Portman and Emmons[172] further indicate that no other bHLH appears to be expressed in the ray sublineage. Taken together, these observations indicate that, in *C. elegans*, a same bHLH protein may be expressed at and may control all steps of a given lineage.

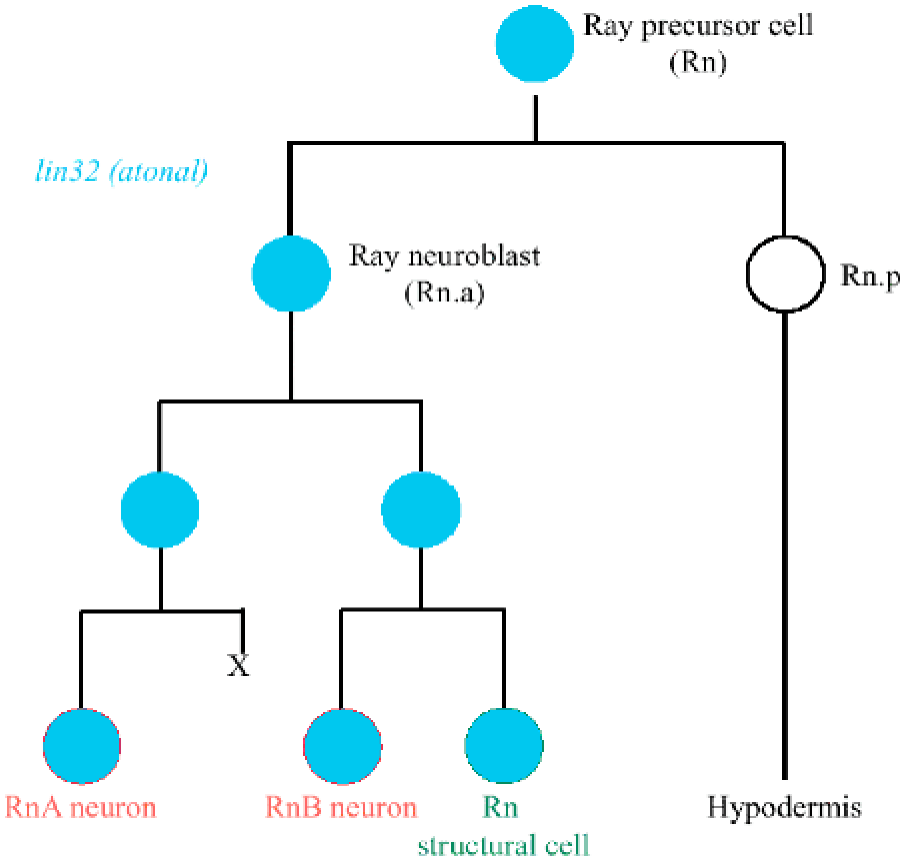

**FIGURE 8.** The ray sublineage in *C. elegans*. The ray precursor cell (Rn) divides and gives rise to an anterior daughter (Rn.a), the ray neuroblast, and a posterior daughter (Rn.p) which gives rise to hypodermis. Rn.a divides twice to generate the two ray neurons (RnA and RnB), the ray structural cell (an accessory cell), and a cell that undergoes programmed cell death (X). The atonal ortholog, lin-32, is expressed in the whole lineage, from Rn to the differentiating daughter cells, except in the non-neural Rn.p cell. No other bHLH is expressed in this lineage.

In the case bHLH proteins act in cascade, i.e., different bHLH proteins acting at different stages, an important question is to determine whether these proteins have different functions. Obviously, they will have different effects, as the cellular context will be different. The important question is whether or not there are functional specializations that are intrinsic to the proteins or whether the different outcomes of the activity of the proteins will solely be due to the different environments. In one extreme model, early and late bHLH (determination and differentiation factors, respectively) would be intrinsically different, i.e., early genes would be unable to fill in late gene functions even if expressed at the right time and vice-versa. Alternatively, in the opposite extreme model, early and late bHLH would be completely interchangeable, their actual function being solely determined by the timing of their expression. There is so far no definitive evidence for one or the other of these models. There is, however, one clear observation that supports the second model — in Drosophila, asense, while not involved in neural precursors formation, is able to rescue LOF phenotypes of the proneural genes achaete and scute[62,63]. GOF analyses of bHLH in Drosophila and vertebrates are also more consistent with the second model — when overexpressed or misexpressed, various bHLH, irrespective of their normal timing of expression, give a largely similar phenotype, i.e., presence of additional neurogenesis[9,61] (see previous sections). The available evidence do support the idea that bHLH expressed at different stages of the neural lineages are largely interchangeable. If this is true, this raises a question: why have several bHLH that would make the same things? We will discuss in the next section the possibility that this is due to subfunctionalization[23], i.e., fragmentation of an ancestral expression pattern that allows preservation of multiple copies after gene duplications. We will further suggest that the ancestral expression and function of bHLH in neural development may be most akin to what is currently observed in *C. elegans*, i.e., expression of a single bHLH throughout a particular neural lineage, which controls various aspects of the differentiation of the neural cells produced by this lineage.

## SUBFUNCTIONALIZATION AND THE EVOLUTION OF NEURAL BHLH GENES

The bHLH families arose from duplications from ancestral sequences that occurred during the early evolutionary history of the eukaryotes[4]. Other duplications have also occurred since the radiation of triploblasts, both in protostomian and deuterostomian lineages. Traditionally, gene duplication is viewed as a way to generate novelties and complexity[173]. Duplicate genes, at the time they arose, are thought to have fully redundant functions, such that one copy may be freed from natural selection (assuming gene dosage is not crucial). As a consequence, the copy that is no more subject to natural selection, accumulates mutations (essentially deleterious) and in most cases, becomes a pseudogene and eventually disappears within a few millions years ("nonfunc-tionalization"). In rare cases, beneficial mutations may occur and endow one copy with a novel function ("neofunctionalization"), while a second copy maintains the initial function[23,173]. This model closely links preservation of duplicated genes with the existence of evolutionnary novelties. Force et al.[23] suggested, based on empirical and theoretical data, that there is a much greater proportion of gene duplicates preservation than expected by the model. They proposed an alternative (but not exclusive) model, the duplication-degeneration-complementation (DDC) model (Fig. 9)[23,24]. This model suggests that the main cause for the preservation of duplicated genes is not the acquisition of new functions (neofunctionalization) but the partitioning of the ancestral function among the two duplicates ("subfunctionalization"). Subfunctionalization would be driven by deleterious mutations that occur in the regulatory region of the two duplicated genes. Given the modularity of eukaryotic regulatory regions, these mutations may do that the two duplicated genes become expressed in complementary subsets of the expression domains of the single ancestral gene. As a consequence, the two duplicated genes are both necessary to ensure the function that was filled in by the single ancestral gene, thereby preventing nonfunctionalization of either of the two copies. Subfunctionalization and neofunctionalization are not mutually exclusive, as subfunctionalization — by facilitating the preservation of the duplicated genes — increases long-term opportunities for the evolution of new gene functions.

One possible example of subfunctionalization in Drosophila is the ASC (Fig. 9). The ASC comprises four related genes that arose from tandem duplications. Three of these genes are expressed in ectodermal patches and control the formation of neural precursors in the PNS and CNS. Their expression ceases before the precursors divide for the first time. The fourth member, asense, is expressed exclusively in already determined precursors and in their progeny (see previous sections). The duplications that generate the ASC occurred rather recently. While clear orthologs of three of the four ASC genes have been found in another dipteran (Ceratitis capitata[174]), a single ortholog to the four ASC genes is found in more distantly related insects, i.e., the buckeye butterfly (Juonia coenia), a lepidoptera, and in the flour beetle (Tribolium castaneum), a coleoptera[175]. Hence, duplication probably occurred after the divergence of diptera from other insects. The expression of J. coenia ASH (JcASH1) is highly reminiscent to that of its Drosophila orthologs but comprises both a proneural phase (ectodermal patches and precursors) and asense-like phase (precursors and their progeny).

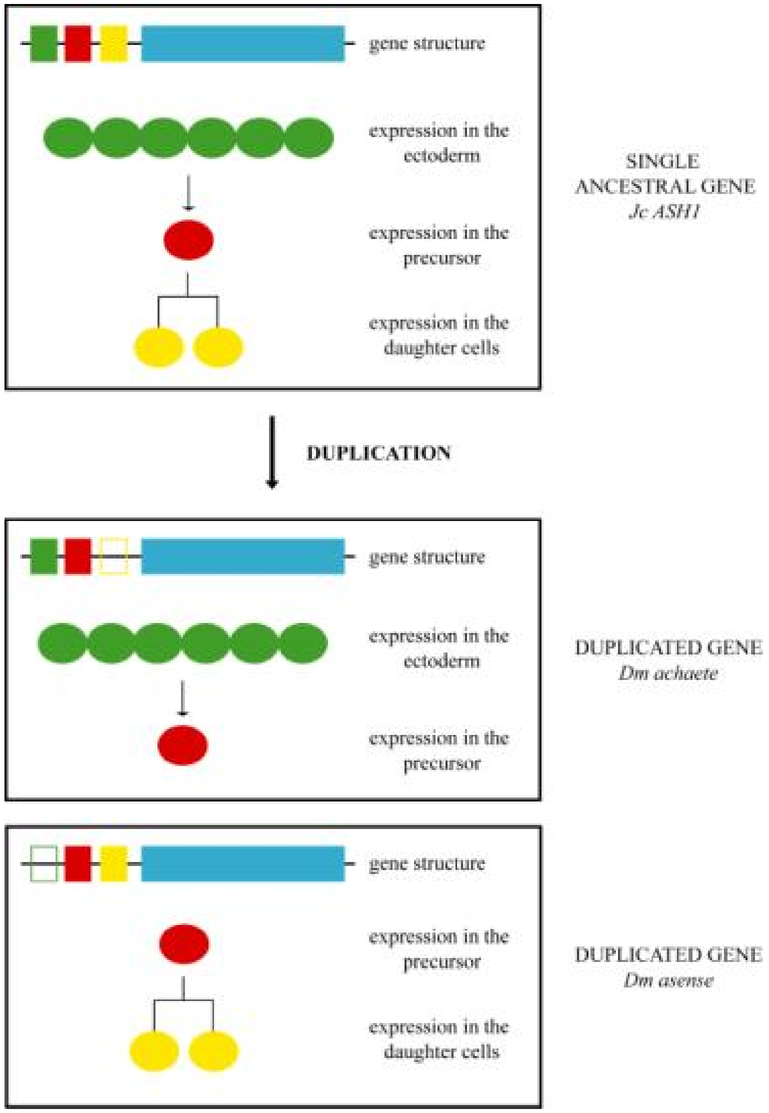

**FIGURE 9**. Gene duplication and subfunctionalization. Two present-day species are shown, one with a single ancestral-like gene, the other with a gene pair resulting from a duplication of the single ancestral gene. The structure of the genes is shown: large blue boxes denote transcribed regions, small boxes represent regulatory elements that direct expression of the gene in particular cells. Solid boxes denote intact functional elements while open boxes indicate mutated nonfunctional elements. The Drosophila achaete-scute complex (here represented by achaete and asense) and its single butterfly ortholog (Jc ASH1 [Juonia coenia ASH1]) are taken as example. These genes have three types of regulatory elements that direct gene expression in the ectoderm (green), neural precursors (red), and daughters of the precursors (yellow), respectively. The single Jc ASH1 gene is expressed in these three cell types and the corresponding regulatory elements are supposed to be intact. In Drosophila, asense and achaete are only expressed in some of these cell types, the nonexpression in the other cell types is depicted as resulting from mutations that inactivate particular regulatory elements. The summed expressions of asense and achaete correspond to the expression of the single Jc ASH1 gene, hence nonfunctionalization of either of the two copies does not occur.

We believe that subfunctionalization may serve as a general framework to explain the evolution of the neural bHLH. The data we have summarized in this review point out that the most of the bHLH

involved in neural development are mainly differentiated by their expression patterns while their coding regions appear to perform very similar functions. Whatever the precise neural subtypes they are

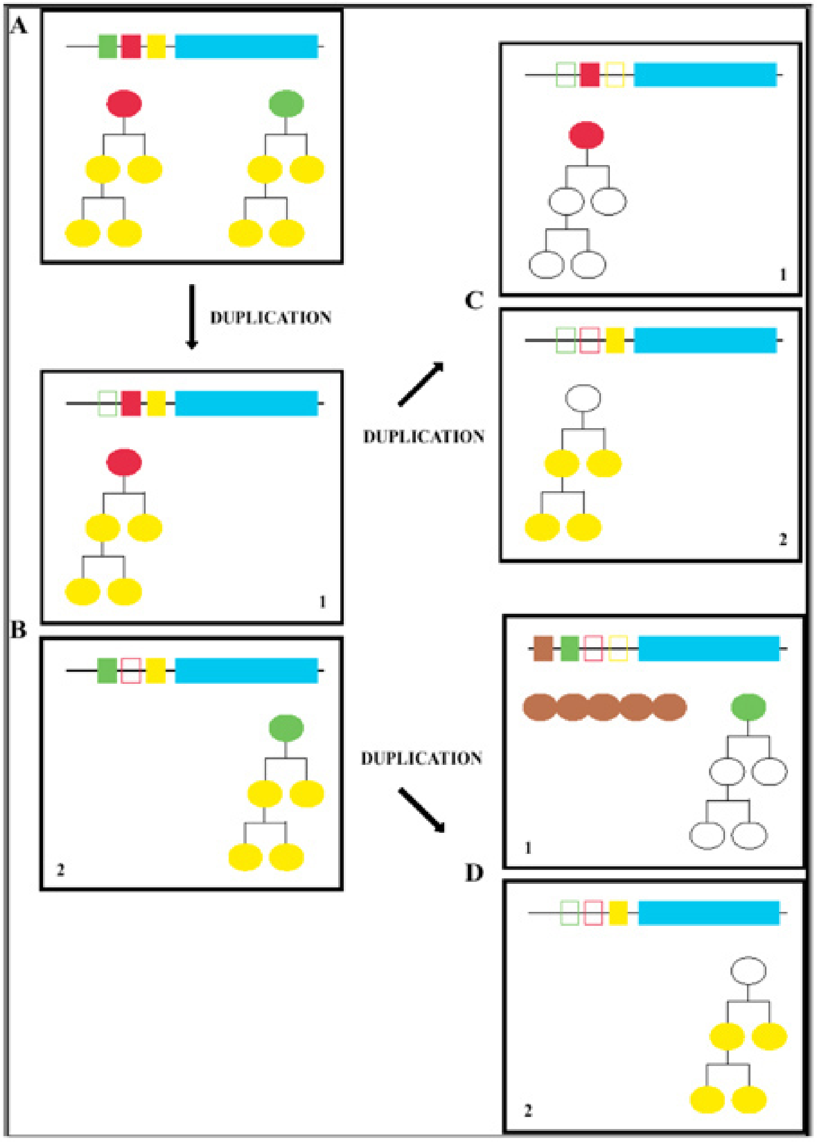

**FIGURE 10.** A scenario for the evolution of neural bHLH genes. (A) The ancestral situation: a single gene is expressed in two different neural lineages both in already-determined neural precursors and their daughter cells. The gene would control the behaviour and the fate of the precursors and their daughter cells, including cell survival and proper differentiation. The expression of the gene in the different cells is controlled by specific regulatory elements (green and red elements direct expression in specific precursors and yellow in the daughter cells). The structure of the gene is schematised as in Fig. 9. (B) A duplication of the gene has occurred, followed by subfunctionalization. The two duplicates are expressed in complementary patterns, the summed expressions of the genes correspond to the expression of the ancestral gene, therefore nonfunctionalization of either of the two copies is prevented. The two duplicated genes show neural subtype specificity as they are only expressed in one particular neural lineage. Nevertheless, the function of the proteins has not been changed and the two genes, therefore, perform identical functions. (C) The situation that may result from the subsequent duplication of one of the genes in (B), followed by subfunctionalization. In this case, the «ancestral» expression pattern has been partitioned such that one duplicate is only expressed in the precursor and the other only in the daughter cells. Again, the two duplicates are required to perform the function of their single ancestor. (D) As in (C). Subfunctionalization by preventing nonfunctionalization of either of the copies allows neofunctionalization to occur: the acquisition of a new regulatory element (brown) allows expression in naive ectodermal cells and, hence, an involvment in neural determination (proneural function). This may have only occurred in the evolutionary lineage that leads to nematodes and arthropods.

expressed in, whatever the precise timing of their expressions, their coding regions appear largely interchangeable. What are then the mechanisms that make these redundant proteins to be preserved from degeneration? We believe it is the fact that the spatial and/or temporal patterns of expression of these proteins are largely different. We propose that these differences primary arose from subfunctionalization with the following scenario (Fig. 10). An ancestral neural bHLH may have had an expression throughout the neural lineages, allowing the correct differentiation of neural cells (including the fact not to form glial cells). This is the function still ensured by single bHLH in nematode or collectively by a cascade of bHLH in vertebrates. A similar function may be found for the cnidarian ortholog of the ASH genes in Hydra, CNASH, which is expressed in the interstitial cell lineage that gives rise to nematocysts[176]. Following gene duplications, subfunctionalization may have occurred, thereby spatially and temporally partitioning the ancestral pattern between the duplicates. This may have resulted in having bHLH expressed in some neural lineages but not in others and/or at some stages of these lineages and not at others, without implying that these specific expression patterns confer any specificity to the neural cells. However, subfunctionalization may have contributed to the developmental isolation of the neural lineages, which may have been a key property for subsequent neural diversification[11]. Furthermore, the presence of numerous duplicates may have allowed acquisition of new features such as new regulatory interactions. In that way, the proneural function of some bHLH in Drosophila could be interpreted in such an evolutionary novelty (Fig. 10). Of course, the model we propose, which focuses on modifications of regulatory regions, does not rule out the possibility that coding regions evolve new or modified functions. Along that line, it is worth mentioning that elegant genetic manipulations in mouse have indicated that, during telencephalon development, MASH1 and Ngn proteins have different intrinsic activities[177]. Moreover, given the modular structure of proteins, the subfunctionalization model presented above may be easily extended to coding regions and may be applied to some aspects of protein function or post-transcriptional regulation.
